# The Statistical Report for 1914

**Published:** 1915-11-06

**Authors:** 


### KING EDWARD'S HOSPITAL FUND.

### THE STATISTICAL REPORT FOR 1914.

Considerable space in these columns has been devoted to the annual volumes of statistics issued by King Edward's Hospital Fund, and a large part of the notice appearing in our issue of January 23, 1915, dealt with the difficulty, from the statistical point of view, of comparing the teaching hospitals with other large general hospitals not having medical schools attached, and the desirability of dividing the larger hospitals into two groups. It is with much pleasure that we notice the editor of the report for 1914 has adopted the alteration advocated. The prefatory address to the Governors of the Fund explains this departure as follows: "On this occasion the results of hospitals to which medical schools are attached are for the first time stated separately from those relating to hospitals working without medical schools; this rearrangement has been made at the suggestion of some of the hospitals, and we think it will now be easier to compare the expenses of hospitals of a similar character with each other, as well as with those which work on different lines."

### Improvements in the Arrangements.

This is but one of the many improvements in the arrangement of the figures that have been consistently recommended in our reviews of the reports. That other changes which experience has shown to be desirable may be introduced is foreshadowed in Mr. H. R. Maynard's preface, where it is pointed out that as the form of the report for 1914 differs from that of previous issues, and the present publication may be taken as the first of a special transitional series, a convenient opportunity occurs for introducing changes of a helpful nature. The chief divergencies from the familiar form of arrangement in the report under notice lie in the omission altogether from the prefatory note of the comparisons between 1914 and the previous year, and in the tables of the figures of relative cost based on comparisons between each hospital in a group and the average of the group.

The hospitals of the Metropolis have shown for 1914 (as will be shown for 1915) considerable variations in respect of the number of occupied beds, as compared with the averages of previous years. In the cases of the hospitals taking charge of military patients, every effort has been made to modify as much as possible the inconvenience to the civilian public. This has been done in one or both of two ways: (*a*) by the introduction of beds extra to the normal establishment into the wards, and (*b*) by the provision of new beds temporarily placed in other departments, such as outpatient halls and elsewhere. With regard to the wisdom of cramping the accommodation of our voluntary hospitals for any length of time we have had something to say; our present duty is merely to regard the matter from the statistical standpoint. The obvious effect in hospitals working under these conditions is to produce an unnatural figure of average occupation, which, being divided into the gross ordinary expenditure, containing as it does many large standing charges, gives a cost, under some headings, of the occupied bed, which for purposes of comparison is so misleading as to render the produced result of little value to any but the student with sufficient technical knowledge to make the necessary allowances. In other words, the very large increase in expenditure, due to high prices of commodities and rates of service, is neutralised, for present statistical purposes, by the inflated divisor caused by the over-number beds.

On the other hand, in respect of the hospitals wherein there are no military patients and no extra beds, we have a large increase in ordinary expenditure, and a normal average occupation in some cases and a lower average in others where the difficulties in obtaining sufficient medical and nursing service has resulted in the closing of wards. Here, as important standing charges remain, whatever the number of beds used, the average cost under most headings is of little value for comparison.

### Increase in Cost.

In this, the Twelfth Annual Statistical Report, the expenditure of 108 hospitals is dealt with (as against 106 in the report for 1913). The gross result of the tabulation shows that there has been a general increase in cost combined with a decrease in the number of occupied beds. Thus the total provision for the civilian in 1914 decreased considerably, and the extra temporary beds alluded to above have, taking the Metropolitan hospitals as a whole, failed to meet the exigencies of the situation. The tables in the report contain particulars of the numbers of naval and military patients, enabling the reader to estimate what proportion of the work of the year is attributable to these cases, and the amount of the accommodation left for use of the ordinary patients. Thus we see that of a total of 10,939 beds in 108 hospitals, 8,963 were in average daily occupation, 308 of these being devoted to sick and wounded sailors and soldiers. By subtracting the figures relative to the two hospitals included for the first time (Salvation Army Maternity Hospital and Stoke Newington Hospital for Women), we are supplied with figures for comparison with the totals for 1913, which were 10,535 beds, of which 9,034 were in daily average occupation. Therefore, the civilian occupation of 106 institutions for 1913 was 9,034, and that for 1914 8,599. For the ordinary users of hospital beds in London in 1914, 435 fewer beds were provided. In studying these figures, it must be remembered that it was only in respect of five months at most in the year that the military beds were in use; and that in some cases of hospitals offering beds to the authorities, the wounded did not begin to arrive until after the close of the year.

